# Loneliness and Self-Rated Physical Health Among Gay, Bisexual and other Men who have Sex with Men in Vancouver, Canada

**DOI:** 10.1136/jech-2019-213566

**Published:** 2020-04-08

**Authors:** Megan E. Marziali, Heather L. Armstrong, Kalysha Closson, Taylor McLinden, Lu Wang, Justin Barath, Marianne Harris, Eric A. Roth, David M. Moore, Nathan J. Lachowsky, Robert S. Hogg, Jordan M. Sang, Kiffer G. Card

**Affiliations:** 1 Epidemiology and Population Health Program, BC Centre for Excellence in HIV/AIDS, Vancouver, Canada;; 2 Mailman School of Public Health, Columbia University, New York City, United States;; 3 Department of Psychology, University of Southampton, Southampton, England;; 4 School of Population and Public Health, Faculty of Medicine, University of British Columbia, Vancouver, Canada;; 5 AIDS Research Program, BC Centre for Excellence in HIV/AIDS, Vancouver, Canada;; 6 Department of Family Practice, Faculty of Medicine, University of British Columbia, Vancouver, Canada;; 7 Canadian Institute for Substance Use Research, University of Victoria, Victoria, Canada; 8 School of Public Health and Social Policy, Faculty of Human and Social Development, University of Victoria, Victoria, Canada;; 9 Faculty of Health Sciences, Simon Fraser University, Burnaby, Canada;

**Keywords:** mental health, psychosocial factors, self-rated health

## Abstract

**BACKGROUND:**

Due to stigma and discrimination, gay, bisexual and other men who have sex with men (gbMSM) potentially carry a heightened burden of loneliness. This analysis investigates loneliness among gbMSM and its’ relationship with self-rated physical health, along with the mediating effect of depression.

**METHODS:**

Participants were recruited using respondent-driven sampling into the Momentum Health Study (February 2012-February 2015) with follow-up visits occurring every six months to February 2018. Using computer-assisted self-interviews, measures of loneliness were assessed using a 6-item Loneliness Scale for Emotional and Social Loneliness (lonely vs. not lonely). Current physical health was self-assessed (poor, fair, good, very good, or excellent). A multivariable generalized linear mixed model with a logit link function was used to examine the relationship between loneliness and self-rated physical health. We further investigated the mediating effect of depressive symptomatology on this relationship, via the Hospital Anxiety and Depression Scale.

**RESULTS:**

Of 770 participants included, we found that 61% (*n*=471) experienced loneliness at baseline. Of the 674 (88%) who reported *good/very good/excellent* physical health, 59% (*n*=391) reported loneliness, compared with 87% (*n*=80) of those in poor/fair self-rated physical health who reported feeling lonely. After adjustment for confounding, loneliness was associated with poor self-rated physical health (adjusted Odds Ratio: 1.71; 95%Confidence Interval: 1.13–2.60). Depressive symptomatology was found to partially mediate this relationship.

**CONCLUSION:**

There may be a need for the integration of social, mental and physical health programming, targeted towards gbMSM, to alleviate the degree of loneliness experienced and its co-occurrence with poor self-rated physical health.

## INTRODUCTION

The need for belonging and social integration is deeply ingrained within human nature, with social connectivity necessary for health and well-being[6]. However, in spite of this basic need for connection and inclusion, rates of social isolation and loneliness are increasing[7]. Though related, social isolation and loneliness are different concepts. Social isolation refers to objective aspects of social contact such as living alone or lacking a partnership, while loneliness is a subjective construct, often described as perceiving discordance between desired and actual degree of social connectivity[8].

Within general population research, loneliness has been associated with a multitude of health behaviours and adverse outcomes[3]. Previous research has proposed that loneliness results in feelings of being unsafe, triggering hypervigilance mechanisms and a decrease in one’s ability to exercise self-control[3]. Potentially stemming from difficulties with regulating behaviours, loneliness has been associated with cigarette smoking,[9] and reduced physical activity[10]. Loneliness has also been noted to influence physiological functioning, impacting: sleep,[3, 11] cardiovascular disease,[11] migraines,[11] gene regulation, and immune response and neuroendocrine function[3, 6]. Loneliness has also been identified as a risk factor for alcoholism[4] and mortality[1]. Further influencing overall health and well-being, associations between loneliness and mental health conditions have been found. An association between loneliness and depression has been identified,[2, 4] with some suggesting that loneliness is a risk factor for the development of depressive symptoms[5, 12], whereas others have posited a bi-directional, reciprocal relationship[4] such that individuals experiencing depression may be more prone to loneliness, and those who are lonely may become depressed due to a lack of social contact. Both models involve depression, highlighting the important role that mental health plays in regards to loneliness and well-being.

Resultant from stigma and discrimination, many minority populations including gay, bisexual, and other men who have sex with men (gbMSM) experience minority stress. The framework of the minority stress model outlines how individuals who identify as sexual minorities experience varying sources of stress, limiting participation in social networks[13–15]. Stressors can be in the form of external events, expectations of these events, or internalized stigmas[15]. This model posits that these stressors ultimately lead to adverse physiological and psychosocial effects through allostatic load: a concept which explains negative health outcomes stemming from repeated exposure to stressful events[15, 16]. It is conceivable that upon experiencing a greater degree of chronic stress due to stigma, physical and psychological health may be negatively affected. Further exacerbating these sentiments is stress faced by individuals at the intersection of multiple stigmas, such as those facing racism and/or discrimination on the basis of living with HIV[17]. The effects of stigma influence both quality and quantity of social relationships, through mechanisms such as coping by avoidance,[15, 17] and increase risk of poor health through external stressors that in turn influence development of mental health conditions[15, 18].

Identifying as a sexual minority may increase resilience, which in turn can be protective against stigma and mitigate the effects of stress[19]. Resiliency can act at multiple levels: the individual, based on personal abilities regarding adapting to stressful circumstances, or community-level, which refers to resources within a community that may offer support[19]. Community connection has been identified as a full mediator in the relationship between stigma and stress among White men who identify as a sexual minority[20]. The lack of social support would place individuals at greater risk of experiencing the negative impacts of stress; this is particularly relevant to lonely gbMSM with limited social networks, who may be prevented from accessing these supports. In order to be able to benefit from community resources, an individual must first identify to some degree with the community[19]. However, internalized heterosexism impacts disclosure of sexual orientation[21], which in turn could affect social participation.

Stigma and discrimination experienced by gbMSM, in particular those living with HIV, potentially influences the degree of loneliness and its impact on physical health[3]. Therefore, this analysis set out to (1) document the prevalence of loneliness experienced by a sample of gbMSM in Vancouver, Canada, and (2) explore the association between loneliness and self-rated physical health. An exploratory sub-analysis was carried out to (3) examine the mediating effect of depressive symptomatology on the association between loneliness and physical health.

## METHODS

### Participants and Recruitment

The Momentum Health Study is a longitudinal sexual health study with gbMSM in Metro Vancouver, Canada[22, 23]. Participants were recruited between February 2012 and February 2015 via respondent-driven sampling (RDS), a chain referral method[24]. Information on the study’s use of RDS has been published in greater detail elsewhere[22, 23]. Briefly, initial participants, or “seeds”, were recruited via community-based organizations and sociosexual networking applications and websites. Once eligibility was confirmed and written informed consent was obtained, seeds were given up to six vouchers to be distributed through their network to other eligible gbMSM. To be eligible for participation, individuals were required to: self-identify as a man, inclusive of trans men, report having sex with another man in the past six months, be over the age of 15 years, reside in the Metro Vancouver area, and be able to complete a questionnaire in English. Participants self-completed a 60 to 90-minute computer-based questionnaire, which captured sociodemographic, socioeconomic, and behavioural information. This was followed by a nurse-led questionnaire where participants provided blood samples for HIV, syphilis, and HCV testing. Participants received an honorarium of $50 CAD or could choose to have their names entered in a draw for a gift card or travel voucher; they received an additional $10 CAD for each peer recruited. Those who completed the first survey were also eligible for participation in the longitudinal study, which involved follow-up visits every 6 months for a maximum of 4 years until February 2018.

Ethics approval for this study was granted by research ethics boards at the University of British Columbia, Simon Fraser University and the University of Victoria (H11–00691).

Among 774 participants who completed an enrollment visit, four did not respond to the question on self-rated physical health at baseline. Of the 770 individuals included in the analytical sample at baseline, 37.5% (*n*=289) were less than 30 years, 44.9% (*n*=346) were between the ages of 30 to less than 50 years, and 17.5% (*n*=135) were greater than 50 years (not RDS-adjusted). The majority (62.5%, *n*=481) had an annual income of < $30,000 CAD, reported having additional education beyond high school (77.0%, *n*=593), identified as gay (84.8%, *n*=653), and were HIV-negative (71.3%, *n*=549). Detailed information regarding descriptive statistics can be found in Table 1.

### Exposure Measure

Loneliness was assessed through the 6-item Loneliness Scale for Emotional and Social Loneliness (LSESL) (study α = 0.77; μ = 2.58; standard deviation = 1.99; sample item: *I experience a general sense of emptiness*)[25]. Previously validated in a sample of gay and bisexual men,[26] this scale features a 5-item Likert response system (definitely no, somewhat no, more or less, somewhat yes, definitely yes). Scores obtained through this scale range from 0 (complete social embeddedness, no loneliness) to a maximum value of 6 (complete loneliness). Each item within the scale is coded as having a value between 0 and 1 (dependent on Likert responses), and the sum of numerical values of the dichotomous item scores is tallied to produce a value between 0 and 6. As done in previous studies, the final sum of this scale was dichotomized; participants with scores of 0–1 were classified as not lonely, whereas those with a score of ≥2 were classified as lonely[27–30].

### Outcome Measure

Current physical health was assessed via the question: *How would you rate your current physical health* (poor, fair, good, very good, or excellent). Responses were dichotomized for ease of interpretation to *poor/fair* and *good/very good/excellent*. For the purposes of this analysis those responding *poor/fair* will be classified as experiencing poor health and those responding *good/very good/excellent* as experiencing good health.

### Confounder Measures

We controlled for potential sociodemographic, socioeconomic, clinical and behavioural confounders. This included age at time of visit (less than 30 years; 30 to less than 50 years; 50 or greater), and annual personal income (< $30,000 CAD versus ≥ $30,000 CAD). We also considered substance use as a confounding measure, due to relationship between substance use and loneliness[31] as well as the association between substance use behaviours and negative health outcomes[32]. Consequently, we adjusted for the following factors pertaining to substance use in the past six months: cigarette (tobacco) smoking, cocaine use, ecstasy use, mushroom use, crystal methamphetamine use, and use of speed. We also adjusted for use of oxycodone and oxycodone/acetaminophen, codeine, and benzodiazepines, used within the past six months without a valid prescription from a physician, was also considered.

Other potential confounders relating to both physical and mental health included: HIV status at baseline, Body Mass Index (BMI; <25 versus ≥25), Alcohol-Use Disorder Identification Test (AUDIT) harmful drinking sub-scale (study α = 0.71; continuous; range: 0–16, high score denotes possible dependence),[33] and the Gay/Bisexual Self-Esteem/Internalised Stigma scale (GBSIS; study α = 0.88; continuous; range: 0–21, higher score indicates lower self-esteem)[34]. Mental health was assessed via response to the 14-item Hospital Anxiety and Depression Scale (HADS) anxiety (study α = 0.84; range: 0–21; score >7 denotes borderline or clinically significant symptoms of anxiety) and depression (study α = 0.79; range: 0–21; score >7 denotes borderline or clinically significant symptoms of depression) subscales[35]. A two-level variable incorporating scale responses was constructed, wherein a score >7 in either subscale resulted in classification as experiencing clinically significant symptoms of anxiety and/or depression.

### Statistical Analysis

Prevalence was determined through calculating the number of individuals experiencing loneliness at baseline through the last date of follow-up (February 2018). Due to the hypothesized association between loneliness and stigma, we regressed the continuous LSESL scale on the GBSIS scale.

A multivariable generalized linear mixed model, with a logit link function, was used to examine the relationship between loneliness and self-rated physical health (*good/very good/excellent* health [good health] vs. *poor/fair* health [poor health]). This mixed model, with random intercepts, was selected to account for both the longitudinal nature of the data and clustering introduced as a result of the RDS. Potential confounders were selected for inclusion in the final model by a backward selection approach which used the relative change in the coefficients for the loneliness variable as a criterion, until the minimum change from the full model exceeded 5%. A sensitivity analysis was conducted, in which the model was fit using the continuous LSESL scale.

After constructing our final multivariable model, we hypothesized that depression was a potential mediator along the pathway between loneliness to self-rated physical health. This hypothesis was informed by previous studies that documented relationships between loneliness and depressive symptoms[2–5] and an association between depressive symptoms and poor self-rated health[36]. We completed a mediation analysis to examine whether depressive symptoms, assessed via the HADS depression subscale, were acting as a mediator between loneliness and self-rated physical health. Appropriate confounders as previously outlined were adjusted for, with the exception of the HADS anxiety subscale. Testing for mediation was done using the Monte Carlo method for assessing mediation[37]. Significance was determined through the Monte Carlo method, Sobel p-value and posterior p-value testing; partial posterior methodology was employed as a higher power alternative to other common testing[38].

## RESULTS

### Descriptive Statistics

Loneliness was experienced by 61% (*n*=471) of the sample overall. Further, 88% (*n*=674) of participants reported being in good health, while 12% (*n*=96) were in poor physical health at baseline (Table 1). While 59% (*n*=391) of individuals in good physical health experienced loneliness, 87% (*n*=80) participants in poor health were lonely. Approximately 50% (*n*=333) of those who reported good health experienced borderline or clinically significant anxiety and/or depressive symptoms, whereas 75% (*n*=70) of those in poor health experienced borderline or clinically significant anxiety and/or depressive symptoms.

The coefficient of determination obtained from regressing the continuous LSESL scale on the GBSIS scale was 0.65.

### Multivariable Generalized Linear Mixed Model

As outlined in Table 2, after adjustment for potential confounders, loneliness was found to be associated with poor self-rated physical health among gbMSM (Adjusted Odds Ratio [aOR]: 1.71, 95% Confidence Interval [95% CI]: 1.13, 2.59). The univariable generalized linear model with a logit link function is presented in the supplementary materials [Supplementary Table 1]. Participants missing information for the selected covariates were excluded from analysis which resulted in a final sample of 760 individuals with 3,976 observation-level visits longitudinally. A sensitivity analysis was carried out in order to test the behaviour of the continuous version of the loneliness scale. We found an association with loneliness and poor self-rated physical health (aOR: 1.19, 95% CI: 1.07–1.32) per one-unit increase [Supplementary Table 2]. The average effect size was not considerably different when using the dichotomized scale versus the continuous scale and the effect on other covariates was minimal.

### Mediation Analysis

**Figure 1.**
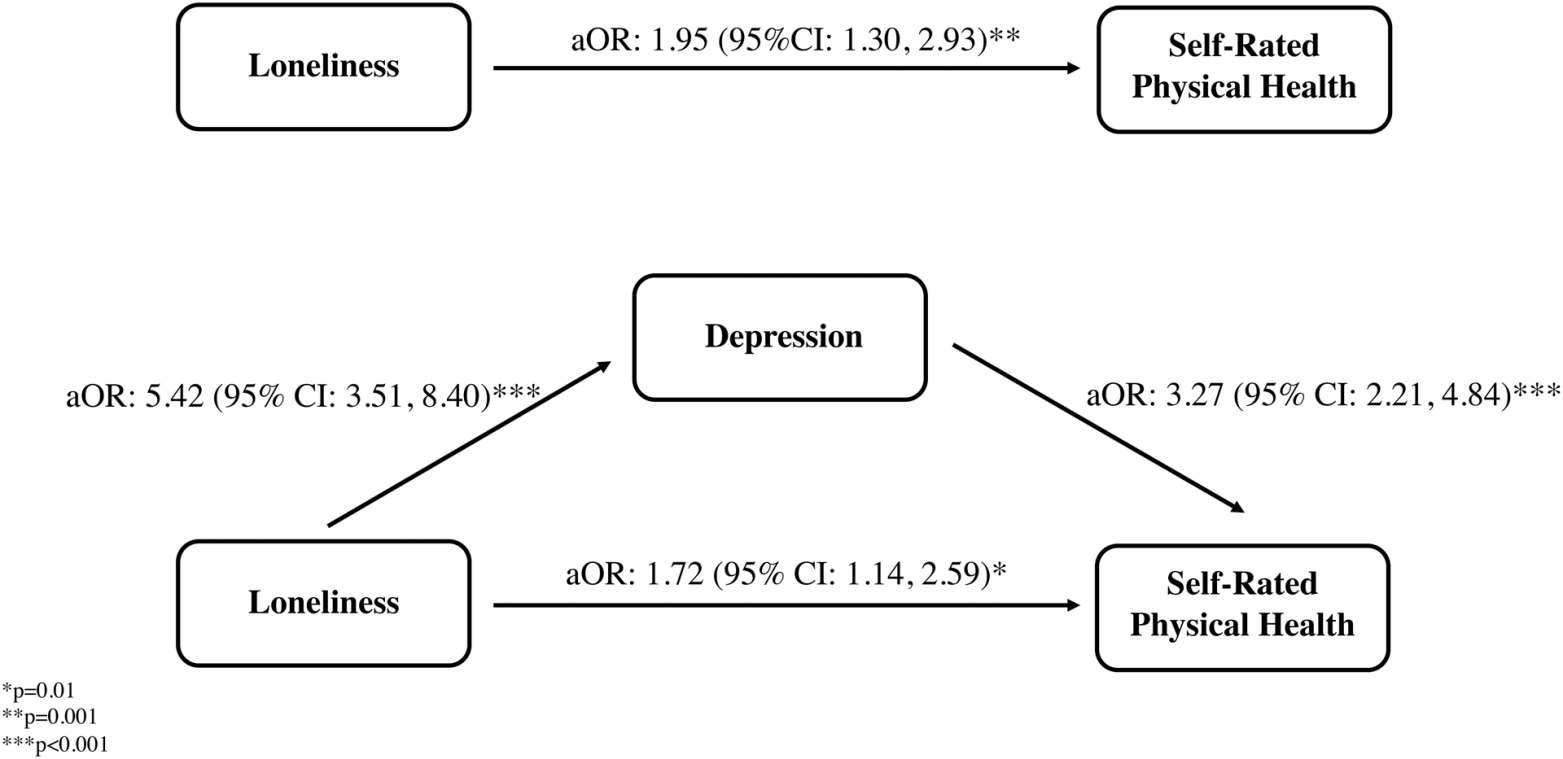
Mediation analysis schematic outlining the ordering between X=Loneliness, assessed through the dichotomized LSESL scale; Y=Self-Rated Physical Health; and M=Depression, assessed through the HADS depression sub-scale, with adjusted odds ratios, 95% confidence intervals and p-values displayed.

Figure 1 illustrates the schematic for the mediation analysis. A higher degree of loneliness was associated with poor self-rated physical health (aOR: 1.95; 95% CI: 1.30, 2.93) without considering the potential mediating effect of depressive symptoms; loneliness was also associated with borderline or clinically significant depressive symptoms (aOR: 5.42; 95% CI: 3.51, 8.40). After adjustment for loneliness, depressive symptoms were associated with poor self-rated physical health (aOR: 3.27; 95% CI: 2.21, 4.84). When adjusting for HADS depression scores as a potential mediator, the magnitude of association between the binary indicator of loneliness and poor self-rated physical health weakened (aOR: 1.72; 95% CI: 1.14, 2.59). We analyzed the proportion of the total effect that was mediated by depression which amounted to 41.5%, suggesting partial, complementary mediation,[39] with both the partial posterior p-value (*P* < 0.001) and Sobel p-value (*P* < 0.001) tests indicating significance.

## DISCUSSION

Our analyses demonstrate a high prevalence of loneliness within the gbMSM population in Vancouver, Canada. The majority (61%) of individuals in this sample experienced some degree of loneliness; 87% of those who perceived their self-rated physical health as poor reported feelings of loneliness. Depressive symptoms partially mediated the relationship between loneliness and perceived physical health, which may suggest that the effect of loneliness on physical health partially operates through depression.

The prevalence of loneliness among gbMSM in our sample can be compared alongside estimates from the general population: roughly 10–23% of populations internationally (e.g. 22% from the United States, 23% from the United Kingdom, and 9% from Japan[40]) reportedly experience some degree of loneliness (ranging from slight to severe experiences of loneliness)[40]. Though a single-item scale was used for the referenced studies, results are comparable[41]. These estimates provide context for the degree of loneliness experienced among gbMSM, and echo findings that outline a greater degree of severity in loneliness reporting among lesbian, gay, bisexual, transgender and intersex (LGBTI) community compared with the general population[42]. The increase in loneliness is potentially attributable to stigma, which may hinder the formation of meaningful social bonds; for example, community members have reported fear of rejection during social interactions can lead to passive coping mechanisms such as avoidance, impacting the quantity of close relationships[14]. Further, bisexual men and women may face greater rejection from their peers in the LGBT community as a result of monosexism, resulting in restricted social connections[43]. This is relevant to discussions within the framework of the minority stress model, which stipulates that the resources a community offers can be beneficial, but communities remain susceptible to enacted stigmas[19]. Experiences of limited social networks may be heightened among men; women and those identifying as transgender within the LGBT community report wider and more diverse social networks[44].

Previous literature has also outlined complex social dynamics among gbMSM, in terms of HIV-related stigma; prejudice resulting in social exclusion, feelings of rejection, and a sense of division has also led towards difficulties forming relationships[45]. Serosorting, wherein men seek out relationships with other men who have the same serostatus, has resulted in feelings of exclusion among men living with HIV[45]. This could contribute towards difficulties forming relationships and thus experiences of loneliness, which highlights a need to target and implement preventative measures to combat loneliness among gbMSM.

Our results suggest a multi-faceted approach wherein loneliness influences self-rated physical health. Previous research has highlighted loneliness as a risk factor for depressive symptoms, with some suggesting these factors are acting synergistically to influence adverse health outcomes[3, 4]. Depressive symptoms were controlled for in the multivariable model; however, we went beyond treating depression as a confounder, and explored this association in detail via construction of a mediation schematic, which allowed us to further delineate the relationship between loneliness, mental health, and self-rated physical health. This is particularly important, as gbMSM have been identified as experiencing poorer mental health outcomes, in comparison to straight men[46]. As depressive symptoms partially mediated this relationship, one avenue through which loneliness potentially impacts health is centred around mental health. Due to the presence of partial and not complete mediation, it can be interpreted that depression is not fully driving the association between loneliness and poor self-rated health. The nature of loneliness and quality of social networks could be influencing access to care when needed, thus impacting health outcomes. Social support has been associated with greater access to care,[47] whereas internalized stigma and non-disclosure of sexual orientation has been found to negatively impact healthcare usage[48]. Loneliness could be impacting health directly through activation of stress-related physiologic responses, through allostatic load[15]. Physical, mental and social pathways could be acting in tandem to contribute towards lower perceptions of overall health.

Regarding limitations, the outcome is based on a single-item self-rated assessment of physical health, which is a subjective measure. Individuals may be rating their physical health lower simply as a result of depression; however, given recent research examining the impact of loneliness on mortality,[1] it seems unlikely that this effect can solely be explained by differences in survey response behaviours. Future research investigating clinical assessments of physical health and loneliness within this population would be beneficial to further elucidate this relationship. It should also be considered that individuals reporting poor physical health may have fewer opportunities for social engagement, due either to discrimination against gbMSM with disabilities or physical and/or emotional limitations. As RDS methodology was used for recruiting participants, it is possible that we have not captured those experiencing severe loneliness. Therefore, the effect size of loneliness on self-rated physical health may be underestimated in this study. Despite our use of longitudinal data, we were limited in our ability to instill a strict temporal-ordering between our measures of loneliness, depressive symptoms, and self-rated physical health in our mediation analysis; results should therefore be interpreted with caution.

## CONCLUSIONS

Our results suggest an association between loneliness and self-rated physical health, partially mediated by depressive symptoms. These findings contribute knowledge regarding the effect of psychosocial factors on the overall well-being of gbMSM and provides insight into the relationship between social, physical, and mental well-being. Acknowledging that community consultation is essential in the development of programming in order to ensure these positive actions will be utilized by the target population, our results provide support for the further examination of whether the development, implementation, and scale-up of programming and comprehensive care that addresses social, physical, and mental well-being is warranted.

## Supplementary Material

Supplemental Table 1 and 2

## Figures and Tables

**Table 1.** Baseline sample characteristics of participants in the Momentum Health Study included in the analytical sample (N=770).

Variable	Outcome	
	Self-rated Physical Health	
	*Good/Very Good/Excellent*	*Poor/Fair*
	*n*=674 *n* (%)	*n*=96 *n* (%)
*Exposure*		
LSESL^1^		
Not lonely	274 (41.2)	12 (13.0)
Lonely	391 (58.8)	80 (87.0)
Missing	9 (0)	4 (0)
*Potential Confounders*		
LSNS		
≥6	637 (95.5)	79 (84.9)
<6	30 (4.5)	14 (15.1)
Missing	7 (0)	3 (0)
BMI		
<25	407 (60.4)	55 (57.3)
≥25	267 (39.6)	41 (42.7)
Mental health condition		
No	335 (50.1)	23 (24.7)
Yes	333 (49.9)	70 (75.3)
Missing	6 (0)	3 (0)
Ethnicity		
White	506 (75.1)	76 (79.2)
Asian	69 (10.2)	5 (5.2)
Indigenous	41 (6.1)	8 (8.3)
Latin American/Other	58 (8.6)	7 (7.3)
Sexual Orientation		
Gay	583 (86.5)	70 (72.9)
Bisexual	53 (7.9)	18 (18.8)
Other	38 (5.6)	8 (8.3)
Transgender		
No	661 (98.1)	94 (97.9)
Yes	13 (1.9)	<5 (2.1)
Age		
Less than 30 years	259 (38.4)	30 (31.3)
30 to less than 50 years	303 (45.0)	43 (44.8)
50 or greater years	112 (16.6)	23 (24.0)
Annual income (Canadian dollars)		
< 30,000	402 (59.6)	79 (82.3)
≥ 30,000	272 (40.4)	17 (17.7)
Baseline HIV status		
Negative	493 (73.1)	56 (58.3)
Positive	181 (26.9)	40 (41.7)
Highest level of education		
High school or less	146 (21.7)	31 (32.3)
More than high school	528 (78.3)	65 (67.7)
Cigarettes^2^		
No	396 (58.8)	45 (46.9)
Yes	278 (41.2)	51 (53.1)
Cocaine^2^		
No	505 (74.9)	66 (68.8)
Yes	169 (25.1)	30 (31.1)
Ecstasy^2^		
No	506 (75.1)	67 (69.8)
Yes	168 (24.9)	29 (30.2)
Mushrooms^2^		
No	599 (88.9)	85 (88.5)
Yes	75 (11.1)	11 (11.5)
Crystal methamphetamine^2^		
No	557 (82.6)	62 (64.6)
Yes	117 (17.4)	34 (35.4)
Speed^2^		
No	637 (94.5)	87 (90.6)
Yes	37 (5.5)	9 (9.4)
Oxycodone, oxycodone/acetaminophen^3^		
No	643 (95.4)	88 (91.7)
Yes	31 (4.6)	8 (8.3)
Codeine^3^		
No	639 (94.8)	87 (90.6)
Yes	35 (5.2)	9 (9.4)
Benzodiazepines^3^		
No	643 (95.4)	85 (88.5)
Yes	31 (4.6)	11 (11.5)
GBSIS (*n*=760, per 1-unit increase) *Median (Q1-Q3)*	*7 (3–9)*	*8 (6–11)*
AUDIT (*n*=765, per 1-unit increase) *Median (Q1-Q3)*	*1 (0–4)*	*1 (0–4)*

LSESL: Loneliness Scale for Emotional and Social Loneliness; LSNS: Lubben Social Network Scale; BMI: Body Mass Index; GBSIS: Gay/Bisexual Self-Esteem/Internalised Stigma scale; AUDIT: Alcohol Use Disorder Identification Test

1 Not lonely (score of 0 to 1); lonely (score of 2 to 6)

2 Use in the past six months

3 Use in the past six months without a valid prescription from a physician

**Table 2.** Multivariable generalized linear mixed model, with a logit link function, quantifying the association between loneliness and self-rated physical health *(n=*760).

Variable	Self-rated physical health (*Good/very good/excellent* vs. P*oor/fair*)	
	Adjusted Odds Ratio	95% Confidence Interval
*Exposure*		
LSESL^1^		
Not lonely	1.00	-
Lonely	1.71	1.13, 2.60
*Confounders*		
Mental health condition		
No	1.00	-
Yes	2.25	1.54, 3.28
Annual income (Canadian dollars)		
< 30,000	1.00	-
≥ 30,000	0.57	0.38, 0.87
Baseline HIV status		
Negative	1.00	-
Positive	2.74	1.53, 4.93
GBSIS (per 1-unit increase)	1.23	1.16, 1.29

LSESL: Loneliness Scale for Emotional and Social Loneliness; GBSIS: Gay/Bisexual Self-Esteem/Internalized Stigma

1 Not lonely (score of 0 to 1); lonely (score of 2 to 6)

## THUMBNAIL SKETCH

### What is already known on this subject

Loneliness is a psychosocial determinant of health, which can lead to adverse health outcomes within the general population, through influencing physiological mechanisms[1]. Further, there is an identified association between depression and loneliness, though the direction of this relationship has not been clearly elucidated[2–5]. It has been well-established that stigma and discrimination exists among gay, bisexual and other men who have sex with men, prompting the authors to investigate the impact of loneliness within this population due to potential heightened vulnerability.

### What this study adds

More than half of the participants in this sample experienced loneliness (61%), suggesting that gay, bisexual and other men who have sex with men are vulnerable to loneliness. Among those who experienced loneliness, 87% reported poor self-rated physical health; after adjustment for confounding, we found an association between loneliness and poor self-rated physical health. Lastly, we determined that depressive symptoms act as a partial mediator along the pathway from loneliness to poor self-rated physical health. These findings highlight the need for assessment of psychosocial stressors to improve health outcomes, and mitigate harm that arises from loneliness.
