# Supplementary material for: Loneliness and Self-Rated Physical Health Among Gay, Bisexual and other Men who have Sex with Men in Vancouver, Canada
Source: J Epidemiol Community Health. Author manuscript; Available in PMC 2021 Feb 1. (PMC7527030; doi:10.1136/jech-2019-213566)
Supplement: Supplemental Table 1 and 2 [file NIHMS1629618-supplement-Supplemental_Table_1_and_2.docx]

Table S1. Univariable generalized linear mixed model, with a logit link function, quantifying the association between loneliness and self-rated physical health (N=760).

| Variable | Self-rated physical health (*Good/very good/excellent* vs. P*oor/fair*) | |
| --- | --- | --- |
|  | Adjusted Odds Ratio | 95% Confidence Interval |
| *Exposure* | | |
| LSESL^1^  Not lonely  Lonely | 1.00  4.09 | -  2.77, 6.03 |
| *Confounders* | | |
| Mental health condition  No  Yes | 1.00  4.49 | -  3.15, 6.39 |
| Annual income (Canadian dollars)  < 30,000  ≥ 30,000 | 1.00  0.51 | -  0.34, 0.77 |
| Baseline HIV status  Negative  Positive | 1.00  3.37 | -  1.74, 6.55 |
| GBSIS (per 1-unit increase) | 1.31 | 1.25, 1.37 |

LSESL: Loneliness Scale for Emotional and Social Loneliness; GBSIS: Gay/Bisexual Self-Esteem/Internalized Stigma

^1^Not lonely (score of 0 to 1); lonely (score of 2 to 6)

Table S2. Multivariable generalized linear mixed model, with a logit link function, quantifying the association between loneliness and self-rated physical health (N=760) with the continuous LSESL scale.

| Variable | Self-rated physical health (*Good/very good/excellent* vs. P*oor/fair*) | |
| --- | --- | --- |
|  | Adjusted Odds Ratio | 95% Confidence Interval |
| *Exposure* | | |
| LSESL (per 1-unit increase) | 1.19 | 1.07, 1.32 |
| *Confounders* | | |
| Mental health condition  No  Yes | 1.00  2.12 | -  1.44, 3.11 |
| Annual income (Canadian dollars)  < 30,000  ≥ 30,000 | 1.00  0.58 | -  0.39, 0.87 |
| Baseline HIV status  Negative  Positive | 1.00  2.61 | -  1.46, 4.65 |
| GBSIS (per 1-unit increase) | 1.21 | 1.14, 1.27 |

LSESL: Loneliness Scale for Emotional and Social Loneliness; GBSIS: Gay/Bisexual Self-Esteem/Internalized Stigma
